# Ovarian Cyst Removal During Cesarean Section: Malpractice or Not?

**DOI:** 10.7759/cureus.80744

**Published:** 2025-03-17

**Authors:** Maria Ntioudi, Ourania Tzirou, Sotiria Triantafyllou, Klearchos Kandylas, Thomas Karagkiouzis

**Affiliations:** 1 Obstetrics and Gynaecology, General Hospital of Giannitsa, Giannitsa, GRC

**Keywords:** cesarean section, intraoperative management, medicolegal implications, ovarian cystectomy, pregnancy

## Abstract

Ovarian cysts are commonly encountered during pregnancy, often being benign and asymptomatic. However, in some cases, surgical intervention may be necessary. This case report presents a patient who underwent ovarian cyst removal during a cesarean section. We discuss the indications for surgery, surgical approach, potential complications, and outcomes. This case aims to contribute to the limited literature on this subject and evaluate whether this practice is justified or constitutes malpractice.

## Introduction

Ovarian cysts are frequently detected during routine antenatal ultrasounds, with an estimated incidence of 1-5.3% in pregnancy [1]. While many cysts resolve spontaneously, some persist, enlarge, or present complications such as rupture, torsion, or suspicion of malignancy [2]. Among those requiring removal during pregnancy or during cesarean section, dermoid cysts are found in 32%, serous mucinous cystadenomas in 19%, endometriomas in 15%, functional cysts in 12%, and paraovarian-paratubal cysts in 6%, with the rate of malignancy amounting to 2% of cases [3]. The diagnosis is generally made during the first trimester via routine ultrasound [4]. Tumor biochemical markers are not always reliable because their values are altered during pregnancy and potentially can confuse clinicians regarding further management [5]. While ultrasound offers satisfactory information regarding the nature of the adnexal mass, MRI can aid in the differential diagnosis and the suspicion or exclusion of possible malignancy [6]. The management of ovarian cysts during pregnancy remains controversial, particularly regarding the optimal timing and approach for surgical intervention [7]. This case report describes the successful removal of a large ovarian cyst during a scheduled cesarean section and examines the surgical considerations, as well as the medicolegal implications of this practice.

## Case presentation

**Figure 1:**
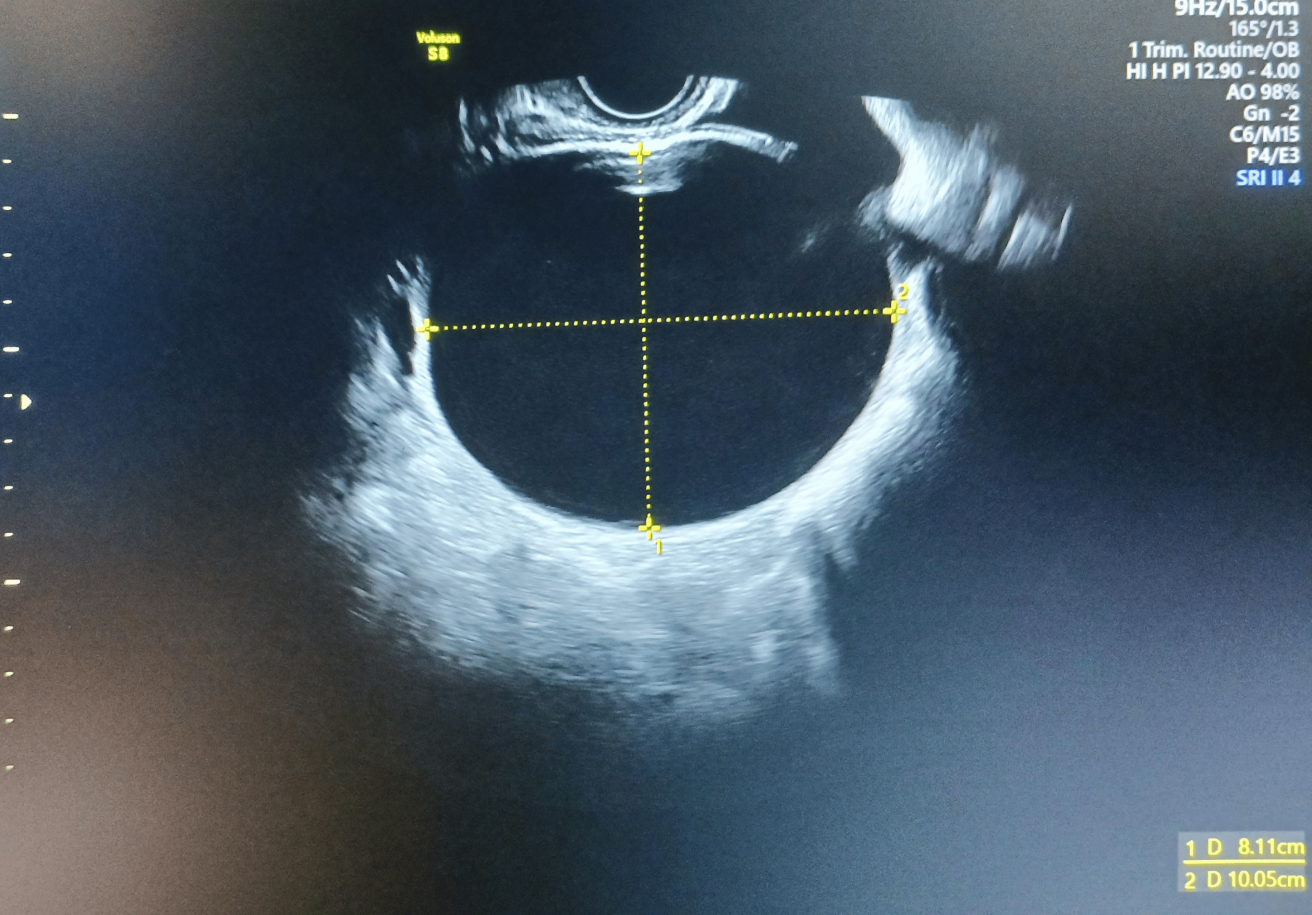
Transvaginal ultrasound exam in the first trimester of pregnancy reveals an ovarian mass.

A 23-year-old primigravida with no significant medical or surgical history was monitored at the outpatient clinic during early pregnancy. A routine ultrasound at 9 weeks of gestation revealed a 10×8×10 cm unilocular ovarian tumor with a thin, smooth wall and serous fluid content (Figure 1).

**Figure 2:**
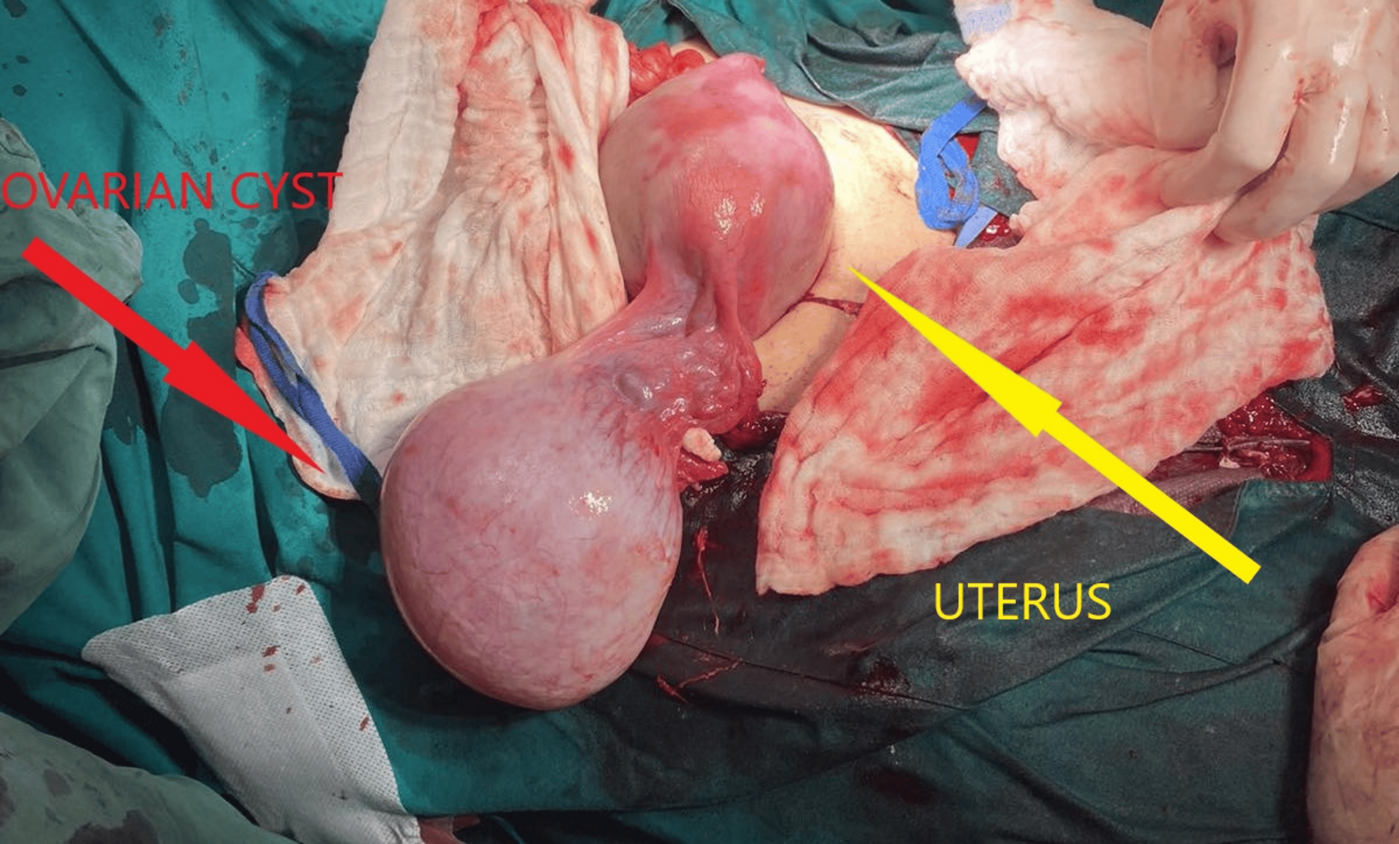
Intraoperative image before the ovarian cyst removal.

Serial ultrasound examinations demonstrated persistence in cyst size without signs of malignancy. It is mentioned that the patient remained asymptomatic throughout the pregnancy. Risk assessment using the International Ovarian Tumor Analysis (IOTA) Simple Rules indicated a low risk of malignancy (<0.5%). Due to the cyst’s persistent size and the emergency cesarean section for failure to progress in labor, intraoperative cyst removal was scheduled with the patient’s informed written consent. A lower-segment transverse cesarean section was performed under spinal anesthesia. After fetal delivery, the right ovary was visualized, revealing an intact, thin-walled cyst. The cyst was carefully excised, preserving the rest of the ovary (Figure 2).

The patient had an uneventful postoperative course and was discharged on day three. Histopathological examination confirmed a benign serous cystadenoma.

## Discussion

The decision to remove an ovarian cyst during cesarean section should be individualized, weighing the benefits against potential risks. Factors such as cyst size, symptoms, risk of malignancy, and the feasibility of surgical removal must be considered. The main concerns with performing ovarian cystectomy during a cesarean section are intraoperative bleeding, risk of rupture, and damage to surrounding structures [8]. Performing an ovarian cystectomy concurrently with a cesarean section offers several advantages. Combining both procedures into a single surgical event reduces overall perioperative morbidity by eliminating the need for a separate surgery. This also minimizes the risks associated with multiple interventions. Additionally, cyst removal prevents future complications such as torsion, rupture, hemorrhage, or neoplastic transformation, which could otherwise necessitate emergency surgery [9]. A single anesthetic event reduces risks associated with prolonged anesthesia exposure, especially in patients with comorbidities.

From a financial perspective, combining the procedures can be more cost-effective, eliminating the need for a second hospital admission, additional anesthesia, and extended recovery. This approach can also result in faster maternal recovery and earlier discharge. Moreover, immediate excision and histopathological evaluation allow for early diagnosis of malignancy, which is crucial for treatment planning and prognosis. However, there are risks involved, such as increased surgical complexity and prolonged operative time. Ovarian cystectomy during a cesarean section can be more difficult, especially in cases involving large cysts, adnexal adhesions, or deeply embedded lesions. This can increase surgery duration, thus raising maternal surgical stress and blood flow complications, which may require blood transfusions or additional hemostatic measures. Furthermore, postoperative complications such as infection, formation of adhesions or hematomas, or delayed recovery can be more likely due to the added complexity of the procedure.

If the cyst is large (>10 cm) [10], adherent, or deeply embedded, there is a risk of unintended ovarian tissue damage or even oophorectomy, which can have implications for future fertility and endocrine function [11]. Literature suggests that ovarian cystectomy during cesarean delivery can be safely performed in selected cases (where the risk of major complications, as mentioned above, is minimized), reducing the need for future surgery and minimizing patient morbidity [12-14]. Thus, while the practice has some clear advantages, a cautious approach is essential, particularly in uncertain histological diagnoses or high-risk cases. Additionally, the medicolegal implications must be considered. Performing an additional procedure without explicit preoperative consent can raise ethical concerns and potential liability issues [15].

The decision to perform ovarian cystectomy during a cesarean section is a complex one, requiring careful consideration of various factors. In the presented case, a 23-year-old primigravida with a 10×8×10 cm benign ovarian cyst, detected early in pregnancy, underwent successful cyst removal during an elective cesarean section. This approach aligns with the literature suggesting that, in selected cases, concurrent cystectomy can be beneficial in preventing future complications such as torsion, rupture, or hemorrhage. The patient’s cyst, which was non-malignant as confirmed by histopathology, posed no immediate threat but had persisted in size throughout the pregnancy, making it an appropriate candidate for removal during the scheduled C-section. By performing the two procedures in a single surgical event, the overall perioperative morbidity was reduced, avoiding the risks of multiple surgical interventions. Furthermore, the cyst’s removal in this instance mitigated the risk of emergency surgery later in the postpartum period. However, this approach requires careful preoperative planning, including informed consent, to avoid medicolegal concerns. Although no intraoperative complications arose in this case, the potential for increased surgical complexity, bleeding, and longer operative times in more challenging cases must be considered. Additionally, while the patient’s postoperative recovery was uneventful, there remains a need for more evidence to confirm the safety and long-term benefits of this practice in varying clinical contexts.

## Conclusions

Ovarian cyst removal during cesarean section is a viable option in selected cases, potentially preventing future complications. However, the question of whether this practice constitutes malpractice or is a justified intervention remains a topic for debate. This case emphasizes the importance of thorough preoperative evaluation, obtaining explicit patient consent, and sound intraoperative decision-making to ensure optimal maternal outcomes and mitigate legal risks. Further research and ethical discussions are needed to establish clearer guidelines for the management of ovarian cystectomy during cesarean section.

## References

[1] Martone S, Troìa L, Luisi S (2021). Adnexal masses during pregnancy: Management for a better approach. Gynecol Surg.

[2] Amant F, Brepoels L, Halaska MJ, Gziri MM, Calsteren KV (2010). Gynaecologic cancer complicating pregnancy: An overview. Best Pract Res Clin Obstet Gynaecol.

[3] Cathcart AM, Nezhat FR, Emerson J, Pejovic T, Nezhat CH, Nezhat CR (2023). Adnexal masses during pregnancy: Diagnosis, treatment, and prognosis. Am J Obstet Gynecol.

[4] Cavaco-Gomes J, Jorge Moreira C, Rocha A, Mota R, Paiva V, Costa A (2016). Investigation and management of adnexal masses in pregnancy. Scientifica (Cairo).

[5] Sarandakou A, Protonotariou E, Rizos D (2007). Tumor markers in biological fluids associated with pregnancy. Crit Rev Clin Lab Sci.

[6] Horowitz JM, Hotalen IM, Miller ES, Barber EL, Shahabi S, Miller FH (2020). How can pelvic MRI with diffusion-weighted imaging help my pregnant patient?. Am J Perinatol.

[7] Aylin P, Bennett P, Bottle A (2016). Estimating the risk of adverse birth outcomes in pregnant women undergoing non-obstetric surgery using routinely collected NHS data: An observational study. Heal Serv Deliv Res.

[8] Horowitz NS (2011). Management of adnexal masses in pregnancy. Clin Obstet Gynecol.

[9] Gül Ö, Oral HB (2021). Management of adnexıal masses recognızed incıdentally durıng the caesarıan: Our 5 years only central experience. Zeynep Kamil Med J.

[10] (2016). Practice bulletin No. 174: Evaluation and management of adnexal masses. Obstet Gynecol.

[11] Balachandren N, Yasmin E, Mavrelos D, Saridogan E (2021). Does ovarian cystectomy pose a risk to ovarian reserve and fertility?. Obstet Gynaecol.

[12] Dede M, Yenen MC, Yilmaz A, Goktolga U, Baser I (2007). Treatment of incidental adnexal masses at cesarean section: A retrospective study. Int J Gynecol Cancer.

[13] Dawood AS (2017). Cesarean section and associated surgeries: Feasibility and surgical outcomes. Women’s Heal - Open J.

[14] Furau C, Gheorghe GF (2015). Ovarian neoplastic cysts found in consecutive cesarean sections: A case report and literature review. J Reproduktionsmed Endokrinol.

[15] Paredes I, Pastrana M, Gordon A, Tan TL (2011). Incidental adnexal mass at Caesarean section - the value of implementing a comprehensive consenting process. BJMP.

